# Paramyxovirus Fusion and Entry: Multiple Paths to a Common End

**DOI:** 10.3390/v4040613

**Published:** 2012-04-19

**Authors:** Andres Chang, Rebecca E. Dutch

**Affiliations:** Department of Molecular and Cellular Biochemistry, University of Kentucky College of Medicine, Lexington, KY 40536, USA

**Keywords:** paramyxovirus, membrane fusion, viral entry

## Abstract

The paramyxovirus family contains many common human pathogenic viruses, including measles, mumps, the parainfluenza viruses, respiratory syncytial virus, human metapneumovirus, and the zoonotic henipaviruses, Hendra and Nipah. While the expression of a type 1 fusion protein and a type 2 attachment protein is common to all paramyxoviruses, there is considerable variation in viral attachment, the activation and triggering of the fusion protein, and the process of viral entry. In this review, we discuss recent advances in the understanding of paramyxovirus F protein-mediated membrane fusion, an essential process in viral infectivity. We also review the role of the other surface glycoproteins in receptor binding and viral entry, and the implications for viral infection. Throughout, we concentrate on the commonalities and differences in fusion triggering and viral entry among the members of the family. Finally, we highlight key unanswered questions and how further studies can identify novel targets for the development of therapeutic treatments against these human pathogens.

## 1. Introduction

**Figure 1:**
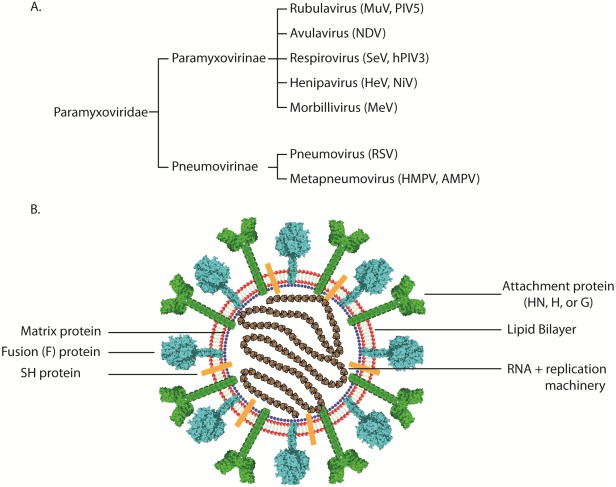
(**A**) Classification of representative members of the paramyxovirus family. (**B**) Schematic of a paramyxovirus. Genomic RNA is wrapped by nucleocapsid core proteins (brown), which are connected to the viral envelope (red) by the matrix protein (blue). The attachment (green), small hydrophobic (present only in certain paramyxoviruses, orange), and fusion proteins (cyan) are depicted at the virus surface.

The paramyxovirus family includes multiple viruses that are of importance to global economics and human health. Among the members of the family are well-known, highly infectious worldwide human pathogens such as measles (MeV), mumps (MuV), and respiratory syncytial virus (RSV), a recently discovered human respiratory virus that is also of global significance (human metapneumovirus, HMPV), and deadly zoonotic viruses such as Hendra (HeV) and Nipah (NiV). Paramyxoviruses also cause disease in other species (such as parainfluenza virus 5 [PIV5] and Sendai virus [SeV]), some of which bring about a tremendous economic burden to society by causing serious, and sometimes fatal, disease in poultry (Newcastle disease virus [NDV] and avian metapneumovirus [AMPV]), cattle (bovine RSV [BRSV]), horses (HeV), and pigs (NiV) [1,2,3,4,5,6]. While these viruses share many common characteristics, such as possessing a negative-sense single-stranded RNA genome and a lipid bilayer envelope [4], there are also many unique aspects in their lifecycles. Based on morphologic criteria, the activity of their proteins, and sequence homology, viruses in this family are divided into seven distinct genera, of which five belong to the *paramyxovirinae* subfamily and the remaining two are grouped in the *pneumovirinae* subfamily. Thus, viruses like RSV and HMPV are classified in the *pneumovirinae *subfamily while MeV, SeV, NDV, the henipaviruses, and the parainfluenza viruses are part of the *paramyxovirinae* subfamily (Figure 1A). Several recently discovered paramyxoviruses such as J virus, Mossman virus, and Salem virus have not yet been classified within a subfamily [4].

Out of the six to ten genes present in the genomes of paramyxoviruses, only two or three of the encoded proteins are expressed at the surface of the virus (Figure 1B). The rest of the encoded proteins are involved either in viral genome transcription/replication or assembly and are beyond the scope of this review. Paramyxovirus entry occurs through virus-cell membrane fusion and is usually mediated by the attachment protein (H, HN, or G) and the fusion protein (F) [4]. To date, there is no evidence to suggest that the third putative viral surface glycoprotein, the small hydrophobic protein (SH), plays a direct role in the virus attachment and entry steps. In fact, the SH protein has only been found in three of the seven genera of paramyxoviruses, and studies suggest that it is not essential for viral infection and replication [7,8,9,10,11].

## 2. Paramyxovirus Attachment Proteins and Viral Receptor Binding

The attachment proteins of rubulaviruses, respiroviruses, and avulaviruses have both hemaglutinin (sialic acid binding) and neuraminidase (sialic acid cleaving) functions and are called HN proteins. Viruses with an HN protein use cellular surface sialic acid as their receptors with various degrees of affinity [12]. The attachment proteins of morbilliviruses (H) lack neuraminidase activity but can bind sialic acid. However, morbilliviruses use cellular proteins, such as CD46, CD150/SLAM, and nectin-4, in the case of MeV [13,14,15], as receptors for attachment. Henipaviruses and members of the *pneumovirinae *subfamily have attachment proteins that do not bind sialic acid and are named G (for glycoprotein). Hendra and Nipah G bind to cellular Ephrin B2/B3 [16,17,18,19] whereas previous reports have shown that *pneumovirinae* G proteins bind to heparan sulfate proteoglycans [20,21]. In addition to mediating the initial attachment of the virus to a cell, attachment proteins of most paramyxoviruses also have fusion promoting activity as coexpression of the homotypic attachment and fusion proteins is required for membrane fusion and viral spread to occur [13,22,23,24,25,26,27,28,29,30]. The cascade of events occurring after receptor binding that leads to the triggering of the F protein, however, remains largely unknown.

All paramyxovirus attachment proteins characterized to date are homotetrameric type II integral membrane proteins [4]. They are made up of a membrane-proximal stalk and a large c-terminal globular head domain anchored by a single-pass N-terminal transmembrane domain [4]. The crystal structure of the globular head domain of several paramyxovirus attachment proteins, including NDV HN, PIV5 HN, PIV3 HN, MeV H, HeV G, and NiV G, has been solved, showing that this domain is composed of four six-blade β-propeller fold monomers arranged in a four-fold symmetry [31,32,33,34,35,36,37]. Substantial evidence suggests that the stalk domain of paramyxovirus attachment proteins is likely a helical coiled-coil that in many cases interacts with and determines the specificity of the fusion protein [13,38,39,40,41]. The latest crystal structures of NDV and PIV5 HN have, in fact, provided further evidence that the stalk domain is a tetrameric coiled-coil as the portion proximal to the head domain was observed [37,42]. For most HN attachment proteins, the binding site to sialic acid is located at the top of the globular head and at the center of the β-propeller fold of each monomer [33]. However, the crystal structure of NDV HN protein shows a second sialic acid binding site located at the dimer interface [43,44]. Inhibitor-based studies and studies of mutant viruses also suggest the presence of a second sialic binding site for PIV3 HN that is important for fusion promotion and not for receptor avidity [45,46,47]. Interestingly, this the second binding site was not detected in the crystal structure of PIV3 HN [34] and a more recent study provided evidence that, similar to PIV1 HN [48], this site in PIV3 HN may be covered by N-linked glycans [49]. Thus, the importance of this second sialic acid binding site is still not fully understood. Though the morbillivirus H and henipavirus G proteins have adapted to bind to their proteinaceous receptors, a site analogous to the sialic acid binding site of HN proteins can be found in these H or G proteins, suggesting that they have evolved from a HN-like protein [35,50,51]. While the binding site for Ephrin B2/B3 in the henipaviruses is also located at the top of the head domain of each monomer [31,36], the binding site in MeV H to CD46/SLAM is located toward the sides of the β-barrel of each monomer [51,52]. 

While multiple reports indicate that the interaction between the attachment and the fusion proteins regulates the fusogenic activity of *paramyxovirinae *virus F proteins, studies have shown that both RSV and HMPV are infectious in the absence of their highly glycosylated G protein [8,11,53,54]. Furthermore, cell-to-cell fusion promoted by the F protein of HMPV occurs without coexpression of HMPV G [55,56]. Recent reports have shown that RSV F interaction with cell surface nucleolin and HMPV F interaction with heparan sulfate proteoglycans can mediate viral binding to target cells [57,58,59]. In addition, it has been shown that β1 integrin plays an important role in promoting HMPV infection [58,59]. These observations suggest that, unlike members of the *paramyxovirinae* subfamily, receptor binding activity for members of the *pneumovirinae* subfamily can occur through the F protein and receptor interactions with the G protein are not essential. Interestingly, RSV produces a soluble form of the G protein that plays a role in immune evasion [60]. Therefore, while the attachment protein is required for viral entry of members of the *paramyxovirinae* subfamily, more studies are needed to determine the precise role of the *pneumovirinae* G protein in viral entry. 

## 3. Paramyxovirus Fusion Proteins

**Figure 2:**
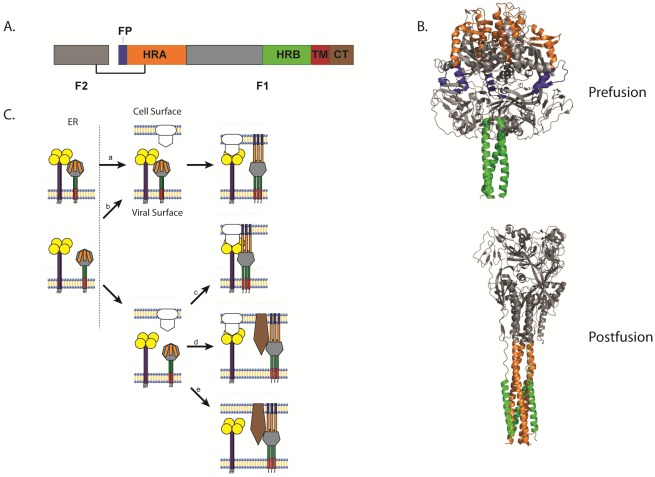
(**A**) Schematic of the cleaved, disulfide-linked paramyxovirus fusion protein. (**B**) Structure of the uncleaved form of PIV5 F in its prefusion conformation [62] and structure of the hPIV3 F in its postfusion conformation [69]. (**C**) Schematic of the different models of paramyxovirus triggering. (**a**) The attachment and F proteins could interact while trafficking through the different biosynthetic compartments and dissociate upon receptor interaction with the attachment protein, allowing the F protein to trigger. (**b**) Alternatively, the attachment and F proteins could travel separately through the biosynthetic pathway, associate at the cell surface, and dissociate after attachment protein interaction with receptor, triggering the F protein. (**c**) The attachment and F proteins could also travel separately and not associate until the attachment protein interacts with its receptor. The association between the attachment protein and the F protein allows the latter to trigger. (**d**) Direct interaction between the attachment and F proteins may not be required for some viruses as receptor binding by the attachment protein could facilitate binding of another receptor by the F protein, allowing the F protein to trigger. (**e**) Finally, some paramyxoviruses do not require the expression of an attachment protein. For these viruses, the F protein binds to its receptor and then promotes membrane fusion, which in some cases can be triggered by low pH. For all images, the fusion peptide is represented in blue, heptad repeat A (HRA) in orange, heptad repeat B (HRB) in green, the transmembrane (TM) domain in red, and brown represents the cytoplasmic tail.

All paramyxoviruses discovered to date express a homotrimeric type I fusion protein. Like other class 1 fusion proteins such as those of influenza, Ebola and HIV, paramyxovirus F proteins have a hydrophobic fusion peptide (FP), two heptad repeat regions (HRA and HRB), are anchored at the surface by a single-pass transmembrane domain (TM), and contain a c-terminal cytoplasmic tail. Paramyxovirus fusion proteins are synthesized as a biologically inactive F_0_ precursor form which must then be cleaved into the fusogenically active F_1_+F_2_ metastable prefusion form (Figure 2A). Upon triggering, the F protein undergoes extensive and irreversible conformational changes that result in the repositioning of the heptad repeat regions to form a stable six-helix bundle (6-HB), a process intimately linked to membrane fusion [4,61].

Analysis of the crystal structure of the uncleaved, GCNt-stabilized PIV5 F protein in its prefusion conformation showed a large globular head domain connected through the HRB-linker region to a membrane-proximal three-helix coiled-coil domain (Figure 2B) [62]. In this metastable state, HRA (orange) is located at the top of the globular head domain and the fusion peptide (blue) is buried between two subunits of the trimer with its N-terminus exposed to the surface for cleavage. HRB (green) is located proximal to the lipid bilayer and is anchored by a TM domain which, in the crystal structure, was replaced by a GCNt trimerization domain. Though the structure of the TM domain is absent from the crystal structure, evidence supports a helical structure which self-interacts [63] and recent studies indicate that TM domains of paramyxoviruses self-associate into trimers and may help stabilize the prefusion conformation [64,65]. Furthermore, several studies indicate that this domain is important for the activity of class 1 F proteins and serves as more than just an anchor to the lipid membrane [63,64,66,67].

Crystal structures of the fusion protein in the more stable postfusion conformation obtained from NDV F [68], hPIV3 [69], and RSV F [70] revealed a strikingly different conformation (Figure 2B). While these proteins remained trimeric in the postfusion state, HRA (orange) forms a new coiled-coil projecting away from the base region of the head domain. HRB (green) translocates to the opposite side of the protein, packed against the HRA coiled-coil creating the characteristic 6-HB. This drastic refolding of the fusion protein creates new intersubunit contacts and disrupts many of those found in the prefusion conformation, creating an overall more compact and stable structure [4]. Recent studies suggest that the FP adopts a helical structure important for fusion [71], and that the FP both associates with itself and with the TM domain [72].

### 3.1. Paramyxovirus F Cleavage Activation

To be fusogenically active, paramyxovirus F proteins must be proteolytically cleaved from the precursor F_0_ form to a disulfide-linked F_1_+F_2_ heterodimer (Figure 2A). Indeed, cleavage of the F protein is an essential step for pathogenicity of the virus. Cleavage activation creates a new N-terminus in F_1_, properly positions the fusion peptide, and may lower the activation energy barrier for triggering [73,74,75]. The cleavage event for most paramyxovirus F proteins occurs during transport through the *trans*-Golgi network, and is promoted by furin, a ubiquitous subtilisin-like cellular endoprotease that recognizes an R-X-K/R-R motif [76]. Cleavage by a furin protease, however, is not ubiquitous for F proteins, as not all paramyxovirus F proteins contain this consensus sequence. Henipavirus F proteins do not express a furin cleavage site and are cleaved after a single basic residue by the cysteine-protease cathepsin L following an endocytic event that brings the F_0_ precursor protein from the surface back to an endosomal compartment [77,78,79]. Furthermore, some F proteins such as those of HMPV and Sendai virus are cleaved by an extracellular protease, a process supplanted in cell culture by the addition of trypsin [53,55,80,81]. *In vivo*, the F proteins of these viruses are likely cleaved by exogenous proteases such as TMPRSS2 and mini-plasmin [81,82]. Interestingly, all paramyxovirus F proteins described to date are activated with a single cleavage event with the exception of RSV F protein, which expresses two consensus sequences for furin cleavage. Cleavage at both sites is necessary for fusion activity of RSV F [83].

The cleavage of the F protein may result in structural rearrangements of the F protein, as peptide antibodies against the heptad repeat regions of PIV5 recognized primarily the uncleaved form of PIV5 F [84]. Furthermore, replacement of the cleavage site in Sendai F with the cleavage sites of RSV F rendered the F chimera capable of promoting syncytium formation in the absence of its homotypic HN protein [75], suggesting that alterations at the cleavage site can affect triggering of the F protein. While some studies with NDV F indicate that cleavage of the F protein is a determinant of virulence, as NDV F proteins with multibasic cleavage sites are more virulent than those with a single basic cleavage site [85,86], a recent study with APMV-2 strain Yucaipa, which lacks the furin consensus sequences but replicates in the absence of exogenous proteases [87], suggests that the cleavage site plays an important role for viral replication *in vitro *but does not alter the virulence of the virus [88]. Therefore, the cleavage event may determine pathogenicity in some paramyxoviruses.

### 3.2. Triggering of Paramyxovirus F and the Role of the Attachment Protein

Because the refolding of the fusion protein from the prefusion to the postfusion conformation is essentially irreversible, it is critical that the fusion protein is triggered at the right place and time. Therefore, the triggering of paramyxovirus F proteins must be temporally and spatially regulated. For the majority of paramyxoviruses, the expression of the homotypic attachment protein is required for F to promote membrane fusion [4]. Studies have shown that the F protein and the attachment protein interact for a number of paramyxoviruses and that these specific interactions are required for triggering [19,22,23,24,26,27,28,89,90]. Interactions between the F protein and the attachment protein have been primarily shown to occur at the head region [25] of F. While many studies indicate that the stalk domain of the attachment protein is responsible for interactions with the F protein [29,39,40,41,89,91], residues in the TM domain [92,93] and the globular head domain [19,94,95] have also been shown to affect F interactions. It is hypothesized that upon receptor binding, conformational changes in the attachment protein alter the interaction with the F protein, signaling the F protein to trigger [32,36,43,89]. However, a recent study indicates that the continuous activation of the F protein by the attachment protein was required throughout the fusion process [90], suggesting a more complex interaction.

For some paramyxoviruses such as NDV and PIV3, it has been shown that the extent of fusion activity is directly proportional to the strength of the interaction between the fusion protein and the HN attachment protein [29,90,96]. It is therefore hypothesized that the attachment protein could be acting as a molecular scaffold that facilitates the triggering of the fusion protein upon receptor binding [4]. However, fusion activity is inversely related to the strength of morbillivirus H and henipavirus G interactions with their respective F protein [26,97], suggesting that the mechanism by which the attachment protein regulates the triggering of the fusion protein is different between viruses. Interestingly, previous studies have shown that MeV H associates with MeV F intracellularly [98]. Therefore, it is hypothesized that the dissociation of the intracellularly-formed F-H complexes at the surface upon receptor binding allows the F protein of MeV to trigger [99]. An intracellular interaction was not detected between HN and F of PIV5and hPIV3 [100], and studies detected either no interaction [101] or extremely low levels of interaction [24] between NDV F and HN. In addition, cotrafficking of Hendra F and G proteins was not observed [102], suggesting that the MeV model for intracellular association is not a universal feature for the paramyxovirus family. 

Triggering of the F protein in the absence of its homotypic attachment protein has been documented for some paramyxoviruses like PIV5 and SeV, although the presence of the attachment protein dramatically enhances fusion activity in these cases [103,104]. Furthermore, mutations in the NDV F, Sendai F, and PIV5 F can remove the dependency on the homotypic attachment protein for triggering [75,104,105,106,107]. Other mutagenesis studies have shown that the HRB-linker region [108,109], portions of HRA [110], and a region in F_2_ that interacts with HRA [111] are all important for the triggering of F, suggesting that multiple regions of the F protein regulate this process. For viruses belonging to the *paramyxovirinae* subfamily, fusion can be triggered by raising the temperature in the absence of the attachment protein [74,112], suggesting that the attachment protein of these viruses helps lower the energy of activation for F triggering to occur.

Interestingly, it has been shown that the fusion protein of wild type RSV and HMPV alone can promote membrane fusion. Furthermore, RSV and HMPV virions remain infectious in the absence of their homotypic G protein [11,53,55,113,114,115]. While most paramyxovirus F proteins promote membrane fusion at neutral pH [4], low pH has been shown to trigger the F protein of some strains of HMPV [55,56]. Mutagenesis studies of low-pH dependent HMPV suggest that the protonation of a critical histidine residue at position 435 plays a large role in the low pH dependency of the F protein [59,109,116]. The low pH dependency of certain HMPV strains and the observation that wild type F proteins of members of the *pneumovirinae *subfamily can trigger in the absence of the attachment protein suggest that the regulation of this process is different between the two subfamilies. Therefore, a precise role for the G protein of *pneumovirinae *viruses is still unknown.

It is apparent that the regulation of F triggering is a complex process and varies between members of the family. For most paramyxoviruses, it is thought that receptor interactions with the attachment protein trigger the fusion protein. While more studies are needed to elucidate the details on the precise mechanism by which triggering is regulated, five models have been proposed for the role of attachment protein interactions on triggering of the F protein. One model, supported by studies of MeV [26,98] and NDV [24], suggests that the fusion and attachment proteins interact at the endoplasmic reticulum during synthesis and trafficking to the cell surface, with the attachment protein potentially holding the fusion protein in its prefusion conformation. After receptor binding, the attachment protein releases the fusion protein and allows it to trigger (Figure 2C, model A). 

Alternatively, the fusion and attachment proteins could travel independently to the cell surface, where they then interact until receptor binding disrupts this interaction and allows the fusion protein to trigger (Figure 2C, model B). This model is supported by studies in HeV and NiV, where fusion activity and F-G avidity are inversely correlated [19,27,97], but HeV and NiV F undergo a unique endocytic recycling process for cleavage [77,78,79], and differential rates of F and G folding in the ER and trafficking through the secretory pathway have been observed [102,117]. Recent studies with hPIV3 show that the attachment and fusion proteins associate before receptor engagement, and this interaction is required beyond the triggering step [28,90]. These observations are in agreement with an earlier study showing that the extent of fusion promoted by hPIV3 F was dependent on the surface density of hPIV3 HN [104] and suggest that the attachment protein actively participates in the entire fusion process.

A third proposed model suggests that the attachment and fusion proteins do not interact until after receptor attachment, with the subsequent interaction between the two glycoproteins allowing F to trigger (Figure 2C, model C). This model is supported by studies with NDV where F-HN interactions are seen only in the presence of receptor, and mutations altering receptor binding of HN decrease F‑HN interactions and fusion [96,118]. In addition, bimolecular complementation studies of PIV5 F and HN showed increased association of the tagged proteins and corresponding increases in fusion, suggesting that much of the F protein was not normally associated with HN [119]. 

Fusion proteins from RSV, HMPV, and other paramyxoviruses that do not require the expression of the attachment protein to induce membrane fusion demonstrate that interactions between the two glycoproteins are not absolutely required for triggering of all paramyxovirus F proteins [55]. For these viruses, interactions between the attachment protein and cell surface proteins such as heparan sulfate proteoglycans [21,120] may facilitate fusion by bringing the two membranes in close proximity, with F subsequently interacting with a cellular receptor (Figure 2C, model D). However, attachment protein interactions with cellular proteins are dispensable for RSV and HMPV, indicating that the F proteins of these viruses are capable of performing the attachment step [59,120,121] (Figure 2C, model E).

### 3.3. Paramyxovirus F-Induced Membrane Fusion

For paramyxoviruses to gain access to the host cell, they must overcome two lipid barriers that separate the genomic contents of the virus from the cytoplasm. Therefore, paramyxoviruses must unite two lipid bilayers during entry, a process that is very energetically unfavorable. This energy barrier is thought to be surmounted in an ATP-independent manner through the irreversible conformational changes of the fusion protein that occur after triggering which provide the energy required to merge the two membranes. Refolding from the metastable prefusion form to the lower energy postfusion state ultimately leads to the creation of a fusion pore allowing the viral contents to be released into the cytosol.

**Figure 3:**
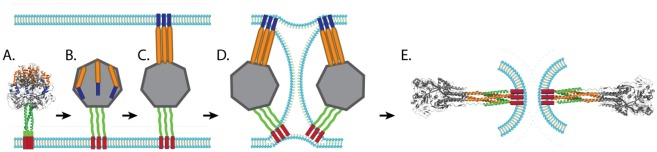
Schematic of membrane fusion mediated by paramyxovirus fusion proteins. After the prefusion form of the fusion protein (**A**) is triggered, the coiled-coil HRB domain melts leading to the formation of the open stalk form (**B**). The FP is then projected towards the target membrane and HRA refolds to a trimeric coiled coil (pre-hairpin intermediate, **C**). HRB then translocates toward HRA, forcing the viral membrane and the target membrane into a hemifusion state (**D**). The complete refolding of HRB around HRA forms the stable 6HB and allows for the formation of the fusion pore (**E**). Blue: FP, Orange: HRA, Green: HRB, Red: TM.

Our understanding of the mechanism of paramyxovirus F protein-mediated membrane fusion has increased greatly over the last decade largely due to the availability of crystal structures of fusion proteins in the prefusion and postfusion conformations. The postfusion F structures of three paramyxoviruses, hPIV3 F [69], NDV F [68], and RSV F [70], have been solved and show that key elements are conserved, such as the formation of a six-helix bundle through the juxtaposition of HRA and HRB. The prefusion structure of PIV5 F [62] is also available and, together with the postfusion structures, suggest that paramyxovirus fusion proteins undergo a “spring-loaded mechanism” of fusion similar to that of influenza HA [122,123]. It is hypothesized that, upon triggering, the HRB coiled-coil present in the prefusion form region melts, creating the open stalk form. Changes in interactions around HRA and the fusion peptide lead to projection of the fusion peptide towards the target membrane and refolding of HRA into a trimeric alpha-helical coiled-coil (pre-hairpin intermediate). Mutagenesis and peptide inhibition studies support the existence of both intermediates, as peptides that mimic HRA can block fusion at an earlier step compared to peptides that mimic HRB [74,108,124]. More direct evidence of the existence of the pre-hairpin intermediate was recently obtained through electron microscopy and computational studies, which confirmed a distance between the membranes consistent with a pre-hairpin intermediate [125]. Changes in the globular head then reposition HRB in an anti-parallel fashion with the grooves of HRA, forming a stable 6-HB and facilitating the opening of the fusion pore (Figure 3). The formation of the fusion pore likely requires the simultaneous refolding of more than one trimeric F protein, as cells expressing PIV5 HN and very low amounts of PIV5 F were able to bind red blood cells without promoting membrane fusion [104].

While the importance of HRA and HRB in promoting membrane fusion has clearly been established, a substantial amount of evidence recently obtained also suggests a regulatory role for the cytoplasmic tails of paramyxovirus glycoproteins. It is thought that cytoplasmic tails transmit a signal to the ectodomain that regulates the conformational changes during membrane fusion, as changes in both the length and sequence of the cytoplasmic tail have been reported to alter the fusogenic activity of the F protein [126,127]. Furthermore, deletion of the cytoplasmic tail of PIV5 F affects fusion pore expansion [128], and F protein chimeras with cytoplasmic tails from other paramyxoviruses also led to changes in F protein expression and activity indicating that the correct sequence needs to be in place for fusion to occur [129]. Interestingly, truncation of the MeV H tail also alters fusogenic activity, suggesting that the cytoplasmic tails of attachment proteins also play a regulatory role in membrane fusion [130].

Recent work on fusion peptides has also shed light on the importance of these membrane-interacting regions in the refolding of paramyxovirus fusion proteins. Evidence to date indicates that fusion peptides are highly conserved between paramyxoviruses and adopt an alpha-helical structure upon contact with membranes [72]. Analytical ultracentrifugation studies have determined that fusion peptides assemble cooperatively into hexamers in a lipid environment [72]. Indeed, mutations affecting the alpha-helical nature of the peptide decrease the efficiency of HeV F protein-promoted membrane fusion [71]. Interestingly, alanine substitution of conserved glycine residues in the fusion peptide of PIV5 F resulted in an increase in fusion activity and a loss of dependency on the HN protein. However, mutations of the same glycine residues in hPIV3 F and NDV F did not allow the fusion protein to promote fusion in the absence of the attachment protein [131]. Furthermore, mutation of the conserved glycine residues altered the expression and processing of HeV F [71]. These observations suggest that these residues may be important in providing stability to the prefusion form and in regulating the kinetic barrier of F protein activation.

More than serving as mere anchors for the ectodomains of paramyxovirus F proteins, TM domains have been shown to play an important role in regulating F triggering and fusion. A glycophosphatidylinositol (GPI) anchored influenza HA protein, another class I fusion protein, does not promote aqueous content mixing in fusion assays, indicating that, for a class I fusion protein, a proteinaceous TM domain is required to promote membrane fusion [132,133]. Interestingly, membrane fusion was abolished when the TM domain of NDV F was replaced with that of MeV F or SeV F even though these mutant proteins were transported to the cell surface and proteolytically cleaved [134], suggesting that either the TM domain is important in triggering, or alterations in the TM affect F ectodomain conformations, resulting in fusion defects. Though structures of paramyxovirus fusion protein TM domains are not available, current evidence suggests that they are composed of interacting alpha-helices [63]. Furthermore, recent sedimentation equilibrium studies showed that the TM domains of several paramyxovirus F proteins self-associate into trimers and that mutations in the GxxxG motif of the HeV F TM domain, known to mediate helix-helix association [135], altered this association leading to a decrease in fusogenic activity [65]. Interestingly, the addition of the HRB domain to the isolated HeV F TM domain destabilized the trimeric interactions between TM domains suggesting that interactions in the head domain and in the TM domain are important for stabilizing the prefusion coiled-coil formed by HRB [64].

## 4. Viral Entry into Cells

Viruses have evolved a variety of mechanisms to gain access to host cells and ensure their survival despite the complex protective machinery implemented by the host. In general, after receptor binding, enveloped viruses enter the target cell either by receptor-mediated endocytosis or through direct penetration at the plasma membrane. A significant amount of knowledge has been obtained on the route of entry for viruses with low-pH dependent fusion proteins such as influenza and the rhabdovirus vesicular stomatitis virus (VSV). These viruses take advantage of the increased acidity of the endocytic pathway to trigger their fusion proteins and deliver their genomic content to the cytoplasm of the cell [136,137]. Additionally, some viruses like Ebola exploit the presence of cellular proteases in these low-pH environments to activate their fusion protein [138].

**Figure 4:**
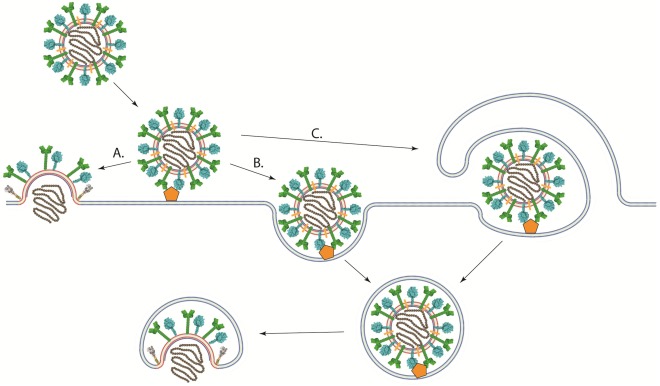
Schematic of different pathways of viral entry. (**A**) Viral-cell membrane fusion of pH-independent viruses could occur at the plasma membrane under neutral pH after binding to receptor. (**B**) A viral particle could enter the cell through an endocytic pathway after receptor binding. Viral-cell membrane fusion could then occur in an intracellular compartment. Low pH may or may not be required for membrane fusion to occur. (**C**) viruses could enter cells through macropinocytosis, where the viral particle is engulfed by the cell after receptor binding. Fusion between the viral and cellular membranes would then take place at an intracellular compartment.

In contrast to viruses with low-pH dependent fusion proteins, viruses that have pH-independent fusion proteins such as most paramyxoviruses and retroviruses have been thought to enter cells at the plasma membrane, where the pH is neutral (Figure 4A) [139,140]. This hypothesis is substantiated by the ability of their fusion proteins to promote syncytium formation when expressed at the cell surface under neutral pH and by infectivity studies in the presence of agents that prevent the acidification of endosomes (bafilomycin and ammonium chloride among others) [116,141]. However, direct evidence of viral entry at the cell surface has not been obtained. Indeed, low pH does not inhibit the activity of the fusion proteins of paramyxoviruses like PIV5 [140], HeV [142], RSV [141], NDV [143,144], and pH-independent strains of HMPV [116]. Furthermore, RSV and NDV fusion, as assessed by a R18 dequenching assay, is enhanced in acidic environments [141,143,144]. Therefore, the pH requirement for fusion does not necessarily clarify the location of the fusion reaction.

Recent studies suggest a more complex mechanism of cell entry for paramyxoviruses. Image correlation spectroscopy studies showed that SeV fusion can occur in the plasma membrane or in intracellular membranes [145]. Other studies using chemical inhibitors, microscopy, and RNAi‑mediated knockdown of proteins involved in endocytosis have shown that multiple paramyxoviruses [79,109,144,146,147] could at least be partially using endocytic pathways to establish infection (Figure 4B). NDV infection was significantly inhibited by agents that sequester cholesterol, and NDV particles were found to colocalize with early endosomal markers, suggesting that NDV may be using the caveolae-dependent endocytic pathway [144]. Despite being largely insensitive to traditional lysosomotropic agents such as bafilomycin A1 and ammonium chloride, RSV infection was significantly decreased when clathrin light chain, AP1B1, dynamin 3, and Rab5A among others players of the clathrin-mediated endocytosis pathway were knocked down [146]. Disruptions of the cellular endocytic and macropinocytic pathways through chemical inhibitors and the expression of dominant negative proteins have been shown to inhibit NiV infection (Figure 4C) [79,147]. Furthermore, we recently reported that infection with the low-pH dependent HMPV strain CAN97-83 was significantly inhibited by treatment with lysosomotropic agents, with the inhibitor of clathrin‑mediated endocytosis chlorpromazine, or with the dynamin inhibitor dynasore [109]. Interestingly, the extent of the effects of lysosomotropic agents on HMPV infection appears to be strain dependent [116]. Therefore, more studies are needed to determine the exact entry pathway for most paramyxoviruses.

## 4. Concluding Remarks

Despite the wealth of information that has been gathered about paramyxovirus F protein-mediated membrane fusion and viral entry, many important questions remain to be answered. Foremost among these is the identification of the triggering signal(s) that are transmitted from the attachment protein to the F protein upon receptor binding. What kinds of conformational changes occur in the attachment protein that signal the F protein to trigger? Where are the interactive sites between the attachment and the F proteins? Are conformational changes and/or interactive sites different between paramyxoviruses so that a homotypic attachment protein is required? While current data suggest that, for some paramyxoviruses, this interaction is between the stalk domain of the attachment protein and the head domain of the F protein [25,37,38,41], the exact location in the F protein remains unknown. Furthermore, the F protein of members of the *pneumovirinae *subfamily can be triggered in the absence of the homotypic attachment protein, suggesting that the triggering mechanism differs significantly between the two subfamilies. 

Important questions also remain on the process of fusion itself. What are the structural intermediates of F in the fusion process? While recent biophysical data have provided us with evidence of the prehairpin intermediate of PIV5 F [125], structural information about the prehairpin intermediate as well as the conformational changes leading to it and after the formation of it remain to be elucidated. Furthermore, the precise role of the attachment protein as an active participant of the refolding of the F protein during fusion beyond the prehairpin intermediate state [90] is still unknown. Are interactions with the attachment protein needed for the F protein to achieve certain intermediate states? If so, what replaces this function of the attachment protein in members of the *pneumovirinae *subfamily?

Another significant area of study is to clarify the role of different cellular pathways in the viral entry process. Increasing amount of data suggest a more complex mechanism of entry that, in many cases, may involve the endocytic machinery of the cell. There are advantages for a virus to enter through an endocytic pathway, as endosomes protect viruses from the host immune system and provide a unique environment for fusion to occur, therefore potentially decreasing the probability of triggering the fusion protein prematurely. However, data to date do not rule out the possibility that paramyxoviruses enter at the plasma membrane or have more than one entry pathway. Lipid mixing and particle uptake do not necessarily correlate with productive infection [148], and thus determining the route of entry for productive infection remains an important goal. 

Recent studies on membrane fusion and entry of paramyxoviruses have provided significant advancements in our understanding of these processes, paving the way for the exploration of potential therapeutic targets using small molecules [149] and peptide inhibitors against the fusion protein [150,151]. While there are indeed conserved regions in the surface glycoproteins that translate into similarities in overall mechanisms of binding and entry, there is a significant degree of diversity in this family that provides for unique aspects of receptor binding, triggering, membrane fusion, and viral entry. Given the importance of this family of viruses to human health and global economy, more studies are clearly needed to better understand both the conserved mechanisms and the unique aspects of paramyxovirus glycoprotein function.
